# Erotomania and phenotypic continuum in a family frameshift variant of AUTS2: a case report and review

**DOI:** 10.1186/s12888-021-03342-8

**Published:** 2021-07-17

**Authors:** Christophe GAULD, Alice POISSON, Julie REVERSAT, Elodie PEYROUX, Françoise HOUDAYER-ROBERT, Massimiliano ROSSI, Gaetan LESCA, Damien SANLAVILLE, Caroline DEMILY

**Affiliations:** 1 Department of Psychiatry, Grenoble Alpes University, Avenue du Maquis du Grésivaudan, 38 000 Grenoble, France; 2 CNRS, UMR 8590 IHPST, Sorbonne University (Paris 1), Paris, France; 3 GénoPsy, Reference Center for Diagnosis and Management of Genetic Psychiatric Disorders, le Vinatier Hospital Center and EDR-Psy Team (National Center for Scientific Research and Lyon 1 Claude Bernard University), Lyon, France; 4 Centre de Biologie et Pathologie Est (CBPE), Hospices Civils de Lyon, Lyon, France; 5 Department of Medical Genetics, Lyon University Hospital, Lyon, France; 6 GENDEV Team, CRNL, INSERM U1028 CNRS UMR5292 UCBL1, Lyon, France; 7 Pôle hospitalo-universitaire ADIS et CRMR GénoPsy, Centre d’Excellence iMIND, CH le Vinatier et Institut Marc Jeannerod, Bron, France

**Keywords:** Autism Spectrum disorder, Erotomania, Persistent delusional disorders, Intellectual deficiency, Phenotypic gradient, *AUTS2*

## Abstract

**Background:**

Pathogenic variants of the *AUTS2* (Autism Susceptibility candidate 2) gene predispose to intellectual disability, autism spectrum disorder, attention deficit hyperactivity disorder, facial dysmorphism and short stature. This phenotype is therefore associated with neurocognitive disturbances and social cognition, indicating potential functional maladjustment in the affected subjects, and a potentially significant impact on quality of life. Although many isolated cases have been reported in the literature, to date no families have been described. This case reports on a family (three generations) with a frameshift variant in the *AUTS2* gene.

**Case presentation:**

The proband is 13 years old with short stature, dysmorphic features, moderate intellectual disability and autism spectrum disorder. His mother is 49 years old and also has short stature and similar dysmorphic features. She does not have autism disorder but presents an erotomaniac delusion. Her cognitive performance is heterogeneous. The two aunts are also of short stature. The 50-year-old aunt has isolated social cognition disorders. The 45-year-old aunt has severe cognitive impairment and autism spectrum disorder. The molecular analysis of the three sisters and the proband shows the same *AUTS2* heterozygous duplication leading to a frame shift expected to produce a premature stop codon, p.(Met593Tyrfs*85). Previously reported isolated cases revealed phenotypic and cognitive impairment variability. In this case report, these variabilities are present within the same family, presenting the same variant.

**Conclusions:**

The possibility of a phenotypic spectrum within the same family highlights the need for joint psychiatry and genetics research.

**Supplementary Information:**

The online version contains supplementary material available at 10.1186/s12888-021-03342-8.

## Background

Genetic diagnosis in neurodevelopmental disorders has yielded a lot of new candidate genes for intellectual disability (ID) and autism spectrum disorders (ASDs) [1]. High-throughput sequencing of intellectual disability/ASD genes identified pathogenic or likely pathogenic variants in 23.5% of patients investigated in day-care hospitals and special schooling institutions [2]. A precise ASD and ID diagnosis is a key challenge as it makes it possible to define the mode of inheritance with a view to assessing the risk of recurrence, and potentially offering a prenatal diagnosis.

*AUTS2* (autism susceptibility candidate gene 2) (OMIM: 615834) maps to 7q11.22, spanning 1.2 Mb of the genomic region (chr7:69,063,905-70,257,885; hg19). The *AUTS2* gene (AUTS2–201, ENST00000342771.10) contains 19 exons coding for a 1259 amino acid protein, and its protein is highly conserved, with amino acid conservation ranging from 62% in zebrafish to 93% in mice and humans [3–5]. *AUST2* is involved in autosomal dominant intellectual disability. It is highly expressed in the neural tube and the embryonic, fetal and adult brain, indicating that it might play an important role during neuronal development [4, 6–8], and more particularly in neuronal migration and neurogenesis [9]. Neuronal migration is one of the pivotal steps to form a functional brain, and disorganization of this process is believed to underlie the pathology of psychiatric disorders including schizophrenia, ASD and epilepsy. *AUTS2* protein has dual functions: cytoplasmic *AUTS2* regulates actin cytoskeleton to control neuronal migration and neurite extension, while nuclear *AUTS2* controls transcription of various genes as a component of the polycomb complex 1 (PRC1) [10]. The set of ID, ASD, neurological abnormalities, and dysmorphic features with short stature, microcephaly and facial dysmorphism has been considered as a syndromic phenotype at the *AUTS2* locus ([3, 6, 11–14]. To date, the findings of numerous studies have provided functional evidence of a causal link between *AUTS2* variations and early growth and neurodevelopment defects [15–17]. AUTS2 syndrome emerges as a specific ID syndrome with dysmorphic features and a specific behavioral phenotype, and most notably with an ASD (MIM 607270).

In the last few years, CNVs involving the whole or parts of the *AUTS2* gene have been reported in more than 100 unrelated patients [14]. Although the existing literature has established a relationship between various psychiatric disorders, including schizophrenia and ASD, and various AUTS2 single nucleotide variants, to our knowledge, only one mutation of two-base pair deletion has been identified in the literature in a male patient with syndromic ID, who has a moderate ID (IQ was tested at 45) with severe language delay and an autism spectrum disorder. This case further supports the finding that AUTS2 syndrome is a single gene disorder [11, 18].

Here we report, for the first time, *AUTS2* locus family transmission of a pathogenic *AUTS2* variant with variable cognitive, psychiatric and social outcomes. As previously documented, the presence of ASD was not constant within the family. This family case report further reinforces the hypothesis of a continuum between ASD and psychosis, including social cognitive disorders.

Here, we report on a psychotic erotomaniac phenotype in the proband’s mother [19, 20]. To our knowledge, this is the first study that suggests a genetic molecular basis for erotomania.

We provide full clinical description of these four patients including treatment outcomes, which may complement the current literature. A specific section on the genetic molecular aspects is provided after these case presentations.

## Case presentation

**Fig. 1:**
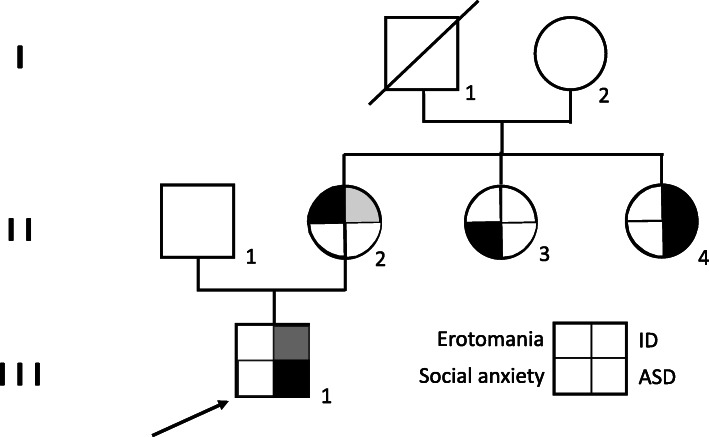
Pedigree analyses of the family carrying the *AUTS2* variant. Level III corresponds to the proband. Level II corresponds to the three sisters. Level I corresponds to the grandparents. The legend describes the correspondence between the phenotype of the members of the family and each part of the nodes. The shading of the nodes conveys the intensity of the ID

Figure 1 shows the family tree of the family studied.

### Proband

The proband is a 13-year-old boy (III1). Pregnancy and birth were unremarkable. Birth weight was 3.3 kg and length 53 cm (5th centile). The mother was 36 years old during the pregnancy. The proband started walking around 2 years old. Significant speech delay was noted by the parents at 3 years of age, but they did not consult for a specialized examination. The child came to medical attention again at the age of 6 due to his global developmental delay and suspected ASD. Indeed, he presented with autistic features such as abnormal behaviors, namely sensory and motor stereotypes (triggered in emotionally charged situations) and compulsive behavior. He has mild-moderate ID (according to the Weschler intelligence scale for children, third edition, WISC-III). In summary, these behavioral findings, along with the developmental delay and dysmorphic features suggest an ASD, according to the Autism Diagnostic Interview, a standardized investigator-based interview, and Autism Diagnostic Observation Schedule, a semi-structured, standardized assessment of social interaction, communication, play, and imaginative use of materials for individuals suspected of having an ASD.

The phenotypic manifestations included craniofacial dysmorphism with highly arched eyebrows, hypertelorism, strabismus, proptosis and downslanted palpebral fissure, thick alae nasi, short philtrum and large central incisors, everted upper lip, narrow mouth, prominent and large ears with uptilted lobules, and retrognathia. His height was 161 cm and weight 78 kg. He has no siblings.

No history of epilepsy was reported. The neurological examination was normal, and no structural abnormalities were observed on the MRI or the EEG. Hearing and sight functions as well as the skeletal X-rays were unremarkable. The urine and blood metabolic screening results were within the normal limits. There was no urogenital or limb malformation. He had no tremors, no picking, no joint stiffness, no feeding difficulties, and no kyphoscoliosis, according to the criteria described by Beunders et al. [3]. He did not have any sleep disorders.

### Mother

The mother of the proband is 49 years old (II2). Pregnancy was uneventful and birth weight was 2.9 kg (5th–10th centile). Her early development was normal, and she walked at the age of around 1 year. Her pubertal development was within the normal limits. The patient had a height of 156 cm (between third and tenth centile) and weighed 76 kg, with a head circumference of 52 cm. The psychological evaluation with the Wechsler Adult Intelligence Scale, third edition (WAIS-III) indicated that her verbal IQ falls within the borderline range (70 to 85). The evaluation of social cognition revealed disturbances in attributional style, theory of mind and emotional recognition. No psychiatric evaluation of a possible ASD was carried out in the absence of clinical signs. She leads an independent life and lives with her husband. No genetic or psychiatric information was available regarding the proband’s father, who exhibited no particular phenotype or behavior.

**Fig. 2:**
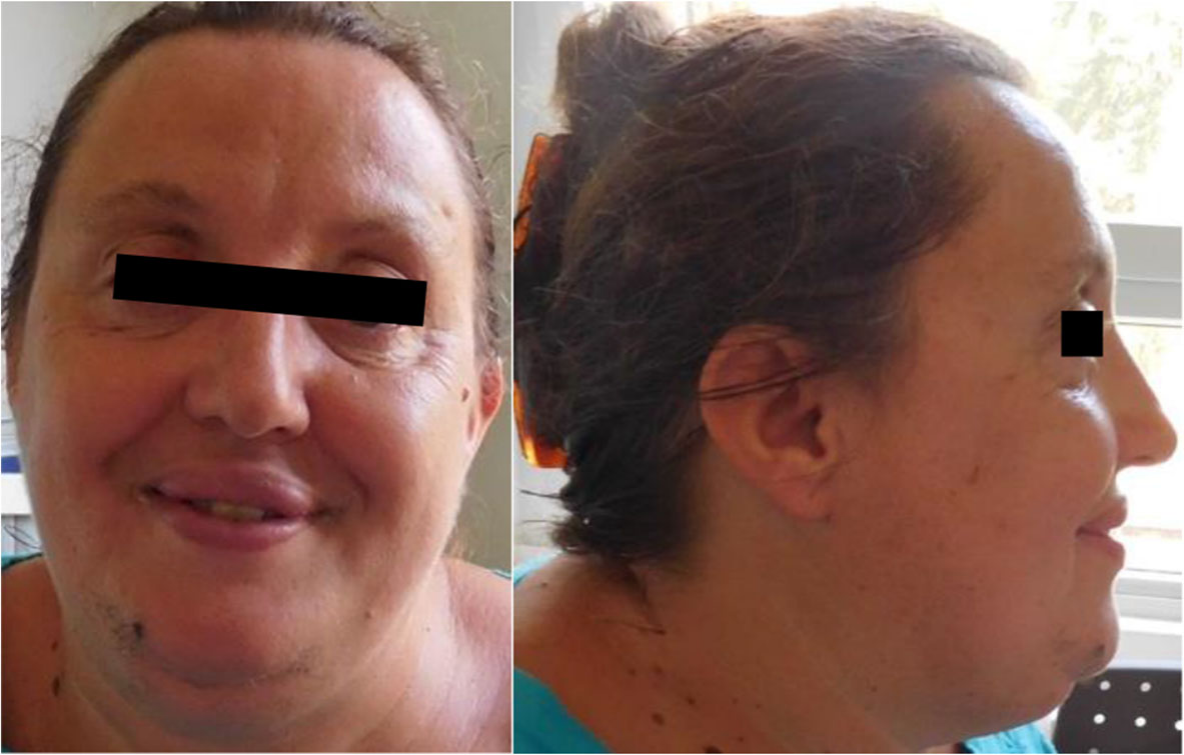
Front and side photographs of the mother showing a heart-shaped face, high broad forehead, short philtrum, slightly posteriorly rotated ears, and arched eyebrows

She presents with short stature, facial dysmorphism with highly arched and thick eyebrows, short philtrum and slightly posteriorly rotated ears. She has arched feet and shallow palmar creases (Fig. 2).

Since the age of 45, she developed delusional ideas with an erotomaniac component. She was convinced that a choirmaster loved her. She was consumed by the idea that every object or drawing she came across were signs that he had left for her as proof of his love. She had a severe interpretive and delusional experience.

The diagnosis of erotomania was made according to the clinical criteria set by the International Classification of Diseases, eleventh edition (ICD-11) (F22.0) and to the Diagnostic and Statistical Manual of Mental Disorders, fifth edition (DSM-5) (297.11). The latter allowed to specify the predominant type of delusional disorder.

There were no urogenital or limb malformations. She had no tremors, no picking, no joint stiffness, no feeding difficulties, according to the criteria described by Beunders et al. [3]. There was no family history of substance-alcohol use. She wore a corset for kyphoscoliosis. She did not present with any sleep disorders or epileptic seizures. Brain MRI and blood screening were within the normal limits.

### Aunt 1

Aunt 1 is 50 years old (II3). Pregnancy and childbirth were unremarkable. Birth weight and birth length are not known. Her early development was unremarkable. The patient has a height of 160 cm (between third and tenth centile) and weighs 78 kg. The psychological evaluation using WAIS-III indicated an IQ within the normal range. The social cognition evaluation did not reveal any disturbances in social cognition but revealed high levels of social anxiety, severely impacting her daily life. She lives in her mother’s home and is not married. She has a job.

This patient presents dysmorphic features such as heart-shaped face, soft fine hair, highly arched eyebrow, a high broad forehead, a bilateral ptosis, large front teeth, kyphoscoliosis and mild micrognathia. No urogenital or limb malformations, nor tremors/picking/feed difficulties/sleep disorders were documented.

Concerning the psychiatric phenotype, Aunt 1 presented serious social anxiety, as has been evaluated during various specialized interviews by several psychiatrists but did not required any medication or supplementary psychiatric evaluations. The blood screening results and brain MRI were unremarkable.

### Aunt 2

Aunt 2 is 45 years old (II4). She was born at term and pregnancy and delivery were both unremarkable. Birth weight and length were not available. The patient has a height of 155 cm (between third and tenth centile) and weighs 61 kg. A lack of eye contact was noted at 6 months of age with global hypotonia and little social interaction. Mild motor and significant speech delay were evident during childhood.

She presents facial dysmorphism with ptosis, minor hand and feet anomalies. Low anterior hairline, narrow sloping forehead, prominent nasal bridge, broad nasal tip, low hanging columella, prominent eyes with bilateral ptosis, arched eyebrows, small mouth orifice, thin upper lip, high narrow palate, micrognathia and kyphoscoliosis were observed, corresponding to the characteristics of the AUTS2 syndrome. She has been followed for scoliosis since early adulthood. She presented feeding difficulties and severe sleep disorder.

She presented mild-to-moderate ID with behavioral problems such as self-harming and aggressivity without psychotic symptoms (verbal IQ < 35, WAIS-III). She had limited eye-contact with severe communication impairment (no verbal language with the use of gestures or grunts to communicate). Psychiatric medication associated Risperidone (2 mg/day), Fluoxetine (20 mg/day), Loxapine (150 mg/day).

### Grandmother and grandfather

The grandmother is 76 years old (I2). We do not have any early information on pregnancy, birth and childhood, which it would appear were within the normal limits. There was notably no language delay. She went to school, worked all her life and apparently never presented any difficulties in her social interactions.

She does not have dysmorphic features. The patient has no abnormal cognitive features, no psychiatric diagnosis has ever been made. Blood screening showed no abnormalities.

As we can see in Fig. 1, her husband (I1) is dead and there is no information available about his birth, development, or cognitive abilities. We learn from the family that he presented qualitative deficiencies in his social interaction and communication.

### Genetic analysis

The patient and the parents of the patient gave their written informed consent before participating in this study, and this study was approved by the ethical institutional review board of Le Vinatier hospital.

#### Proband

The karyotyping result for this patient was 46, XY. Array comparative genomic hybridization (CGH) did not show any unbalanced chromosomal rearrangement. The patient was investigated with a diagnostic gene panel of 450 genes whose mutations cause Mendelian neurodevelopmental disorders, including intellectual disability and autism spectrum disorder. Such a 450-gene panel for intellectual deficiency showed a heterozygous duplication of a single nucleotide, Chr7(GRCh37): g.70236569dup, ENST00000342771.4: c.1769dup in exon 11 of *AUTS2*, leading to a frame shift expected to produce a premature stop codon, p.(Met593Tyrfs*85). No additional pathogenic or likely pathogenic variant was found in any of the other genes of the panel. The history, clinical and behavioral findings support the recurrent phenotypical features of “AUTS2 Syndrome”.

Sanger sequencing showed that the *AUTS2* variant was found, in the heterozygous state, in the mother, aunt 1 and aunt 2 but was absent in the grandmother.

All neuropsychological evaluations were performed by trained neuropsychologists (EP and FH).

Table 1 summarizes the characteristics of the five members of the family carrying the *AUTS2* variant.

**Table 1:** Summary of characteristics of the five members of the family carrying the *AUTS2* mutation

Cases (age)	Development	Phenotype	Neurocognitive features	Social cognition characteristics	Paraclinical parameters	Genetic alteration involving ***AUTS2***
**Proband** **(13)**	Walking at 2 years old Significant speech delay at 3 years old Global developmental delay and suspicion of ASD at 6 years old	Highly arched eyebrows Hypertelorism Strabismus Proptosis Downslanted palpebral fissure Thick alae nasi Short philtrum Large central incisors Everted upper lip Narrow mouth Prominent and large ears Retrognathia	Mild-moderate ID ASD	Qualitative anomaly in interactions and social communication Restricted, repetitive and stereotyped behavioral disorders	MRI normal EEG normal Hearing and sight functions normal Skeletal X-rays normal Urine and blood metabolic screening normal No urogenital or limb malformation	Duplication of exon 11–19 p.(Met593Tyrfs*85) (Chr7(GRCh37): g.70236569dup, ENST00000342771.4: c.1769dup in exon 11) CGH-array: no unbalanced chromosomal rearrangement
**Mother** **(49)**	Early development normal Walking at about 1 year Pubertal development normal (menarche at age 13)	Highly arched and thick eyebrows Short philtrum Arched feet Shallow palmar creases	IQ in the borderline range (70 to 85)	Erotomania (persistent delusional disorder) Strong social anxiety	Brain MRI normal Blood screening normal No urogenital or limb malformation	
**Aunt 1** **(50)**	Early development unremarkable Walking before 2 years No language disorders	Heart-shaped face Soft fine hair Highly arched eyebrow High broad forehead Bilateral ptosis Large front teeth Mild micrognathia	IQ in the normal range	Strong social anxiety	Brain MRI normal Blood screening normal No urogenital or limb malformation	
**Aunt 2** **(45)**	Lack of eye contact at 6 months of age Hypotonic with few spontaneous social interactions Mild motor and significant speech delay after 3 years of age	Narrow sloping forehead Arched eyebrows Ptosis of eyelids Prominent nasal bridge Low hanging columella Small mouth orifice Thin upper lip Micrognathia Scoliosis Bilateral short fifth metacarpals Pes planus	ASD verbal IQ below 35 points: serious intellectual retardation	Limited eye-contact No access to verbal language Gestures or grunts to communicate	MRI normal EEG normal Hearing and sightl functions normal Skeletal X-rays: kyphoscoliosis Urine and blood metabolic screening normal No urogenital or limb malformation	

## Discussion and conclusions

In the present report, c.1769dup of the *AUTS2* gene results in a frameshift. This stop codon reading prevents interaction with a group of Polycomb proteins known to maintain gene expression patterns established during development by regulating the differentiation, proliferation, and maintenance of pluripotency. To our knowledge, this is the first report of this probable pathogenic variant. Thus, this paper presents the first whole family carrier of an *AUTS2* mutation. The dysmorphic features, although of varying severity, were quite similar throughout the family and correspond to the usual phenotype for AUTS2-related syndrome, reported by Beunders et al. [3, 11, 18] and Zhang et al. [21]. In particular, craniofacial dysmorphism including hypertelorism, downslanted palpebral fissure, short philtrum and large central incisors, prominent and large ears, and retrognathia were found in the proband and aunt 2. However, the psychiatric characteristics varied throughout the family. This case seems important in the light of the current literature because it highlights the continuum between different psychiatric disorders, even though only one AUTS2 nucleotide variant has been reported.

The medical findings for the family were summarized and compared to the previously published literature on *AUTS2* gene disruption (see Table 2).

**Table 2:** Clinical review of the literature

Authors	Sultana et al. [5] (on twins of de la Barra et al. [22]	Kalscheuer et al. [23]	Bakkaloglu et al. [24]	Huang et al. [25]	Nagamani et al. [13]	Jolley et al. [12]	Amarillo et al. [6]	Liu et al. [26]	Schneider et al. [27]	Fan et al. (2015) [14]	Beunders et al. [11]	Beunders et al. [18]	Sengun et al. [28]	Saeki et al. (2019)	This report
**Group / family**	Monozygotics twins	Unrelated	Indiv.	Indiv.	Unrelated (2); Siblings (2)	Indiv.	Indiv.	Indiv.	Indiv.	Unrelated	Unrelated	Unrelated (11) et family (2)	Indiv.	Indiv.	Family
**Number**	2	3	1	1	4	1	1	1	1	3	2	13	1	1	4
**Sex**	F (2/2)	M (2/3), F (1/3)	M	M	M (1/1), F (2/3)	M	F	M	M	M (1/3), F (2/3)	M (2/2)	M (5/13) F (8/13)	F	M	M (1/4), F (3/4)
**Mean age (years) and standard deviation**	16 (0)	4, 27, 17	4.5 (0)	4.7 (0)	10 3 10 3	13 (0)	4.5 (0)	4	8.4 (0)	8 8.5 6	20, 24	6 16 7 28 40 23 5 10 59 38 5 1?	68 (0)	8 (0)	13 45 49 50
**Genetic alteration involving** ***AUTS2***															
**Deletion / Duplication / Mutation**	Breakpoint between exons 2 and 3 t(7;20) (q11.2;p11.2) t(7;20) (q11.2;p11.2)	Breakpoint between exons 2–5; 5–6; 5–7 (Transloc.)	Breakpoint within intron 5 inv. (7)(q11.22q35);inv. (7)	Breakpoint within intron 1 (Transloc.)	Deletion of exon 6–14(×2); and duplication of exon 5(× 2) Size: 133 to 319; 179 (2)	Deletion of exon 3–6 o.(Gln107*) Size: 683–806	Deletion of exon 6 Size: 62	Distal deletion of exon 1 Size: 830	Breakpoint between intron 2 and 6, size 89 (Transloc.)	Deletion of exon 6(×2); 12–19(×1), Size: 98; 2147; 262	Deletion of exon 7; 6 p.(Lys286fs) / chr7.hg 19:g.(69985843_69991859)_(70,221,259_70,228,020)del	Deletions of exon 2–4 (× 2); 5(×3); 1–5(× 1); 6(× 2); 6–9(× 1); 9 fs(× 1);7st(× 1); 15–17; all deleted Size: 50 to 4.5 Mb	Deletion of exon 6 and flanking introns 5 and 6 Size: 257	Deletion of exon 8 (p.Tyr488*) (3b)	Duplication of exon 11–19 p.(Met593Tyrfs*85)
**Inherited / De novo**	De novo	De novo (3)	De novo	De novo	De novo (1) Inherited (2) Parent not available (1)	De novo	De novo	De novo	De novo	De novo (3)	De novo	De novo (9) Not available (2) Inherited (2)	Not available	De novo	Inherited
**Neurodevelopment**															
**Developmental delay**	2/2	1/3 (2/3 na)	1/1	1/1	4/4	1/1	1/1	1/1	3/3	3/3	2/2	+ (9/13)	1/1	1/1	2/4
**Intellectual Deficiency**	Severe (2/2)	Severe (1/3) Borderline (1/3) Moderate (1/3)	1/1 (type na)	na	DQ: 5-year-old at 10 (1/4) Vocabulary: 3 words at 3 (1/4) Mild-moderate (2/4)	Intellectual disability (na)	na (“no full sentances”)	DQ: significantly delayed; Developmental age: 11 months (at 4 years old)	Developmental age: 4 years (at 8 years	IQ below 40 (2/3) IQ below 45 (1/3)	Moderate intellectual disability, IQ 45 (1/2) IQ between 60 and 70 (1/2)	Mild borderline (5/13) Mild (3/13) Moderate (5/13)	Mild-Moderate	1/1 (type na)	Severe (1/4) Mild-Moderate (1/4)
**ASD**	2/2	?	1/1	1/1	2/4 (related)	0/1	?	1/1	2/3	2/3	2/2	10/13	1/1	1/1	2/4
**ADHD**	na	2/2, na	na	na	na	0/1	–	1/1	3/3	3/3	1/2	7/13	1/1	1/1	–
**Neurological disorders**															
**Generalized hypotonia**	na	3/3	1/1	–	4/4	1/1	–	1/1	1/3	1/3	–	5/13	1/1	–	2/4
**Structural brain anomaly**	- (EEG)	1/1, na (2)	1/1	na	0/2; na (2)	0/1	–	–	1/3	1/3	–	1/13	–	–	–
**Hypertonia/spasticity**	1/2	1/3	na	–	na	na	1/1	1/1	–	–	–	7/13	na	–	–
**Epilepsy**	2/2	1/3	–	–	1/4	- (one seizure)	–	Na (EEG normal)	2/3	2/3	na	2/13	–	na	–
**Growth**															
**Low birth weight < p3**	0/2	–	–	–	na	–	–	–	–	–	1/2	–	na	1/1	–
**Short stature < p10**	2/2	3/3	–	–	1/3; na (1)	1/1	–	1/1	2/3	2/3	1/2	12/13	1/1	1/1	4/4
**Microcephaly < p2**	1/2	na	na	na	1/4	1/1	–	–	1/3	1/3	2/2	10/12	1/1	**1/1**	**–**
**Feeding problems**	na	3/3	1/1	–	na	1/1		na		2/3	2/2	11/13	1/1	1/1	1/4
**Sleep disturbance**	na	1/3	na	na	na	na		na		na	na	1/13	na	na	1 or 2/4
**Dysmorphic features**	?	+ (2/3)	–	+ (1/1)	+ (3/4)	+ (1/1)	–	–	+ (3/3)	+ (3/3)	+ (2/2)	+	+ (1/1)	+ (1/1)	4/4
**Skeletal abnormalities (Kyphosis/scoliosis or feet deformities)**	+ (2/2)	+ (1/3)	na	na	1/4	na	+ (1/1)	–	1/3	1/3	+ (2/2)	+ (5/13)	+ (1/1)	- (1/1)	+ (4/4)

The number in brackets indicates the number of cases for each study. fs: frameshift mutation; st: stop mutation; Size /kb; *Transloc*. Translocation; *Del*. Deletion; *Dup*. Duplication; *EEG* Electroencephalography; (*nb*) number of bases; *na* not available

Previous papers have found the cognitive and neurodevelopmental outcomes to be relatively constant between members of the same family. For example, Nagamani et al. [13] reported the case of two siblings with ASD, developmental delay, and dysmorphic features. Beunders et al. [3] reported cognitive phenotype in two parents/17 reported cases: one parent had learning difficulties and the other had a mild ID. Our report contradicts this finding. With the same variant (c.1769dup), we found considerable psychiatric phenotypic variation (without phenotypic gradient however) ranging from learning disorders, disabled social cognition, and ID to ASD. The proband and aunt 2 share an ASD. Aunt 2 presents an ID while her two sisters present with a milder phenotype with only social anxiety and social cognition deficits. As previously stated, this variability has been observed in other cases of familial deletions [29, 30], but has never been reported for the *AUTS2* single nucleotide variant, probably because few family cases have been reported.

The early identification of social cognition or neurocognitive disorders may be difficult in some family members. If a genetic diagnosis is already established within the family, close monitoring of the development of other family members would be a legitimate proposition. This accurate, predictive characterization allows for the provision of clinical, socio-professional and medical care at the earliest possible stage. Of course, we are not suggesting proposing genetic explorations for every case of erotomania, but rather that genetic analyses could be offered based on the specific family in question and their environment.

More generally, the psychiatric literature has already reported a broad cognitive phenotype in relation to different pathogenic variants of *AUTS2*. Numerous association studies (Genome Wild Association Studies) have linked *AUTS2* to autism [24, 25, 31], bipolar disorder [32], attention deficit-hyperactivity disorder [33], alcohol dependence [34], heroin dependence [35], epilepsy [36], dyslexia [37], and differences in information processing speed [38].

Schizoaffective disorder was reported by [39]. In 2014, Zhang et al. hypothesized that *AUTS2* polymorphism might be associated with psychosis (rs6943555). Therefore, only a few studies have reported a mutation of *AUTS2* with psychotic phenotypic expression. As far as we know, this is the first report of autistic and psychotic features underpinned by the same *AUTS2* pathogenic nucleotidic variant, therefore assuming a continuum between ASD and persistent delusional disorders. Indeed, as we will detail below, erotomania is a form of persistent delusional disorders [40].

Erotomania is a rare condition. Its prevalence is estimated to be around 15 cases per 100,000 [41], with a sex ratio of 3 women to 1 man (3:1). According to the DSM-5 and the ICD-11, erotomania falls within the scope of persistent delusional disorders (without disorganization: “paranoid delirium with an erotic theme” and should then be distinguished from the diagnosis of schizophrenia) [19, 20]. The patient has the delusional belief that a person has fallen in love with him or her. This belief results from an intuitive mechanism at the beginning, then an interpretative mechanism [42]. It is described that subjects with erotomania usually have low self-esteem and often experience social isolation. They describe emotional excitement during the delusional phase. The social cognitive disorders reported in erotomania correspond mainly to a deficit of theory of mind and an affective style disorder [43].

To our knowledge, the only genetic study of erotomania reported in the international literature was a twin study by Farmer et al. showing less heritability for erotomania than schizophrenia [44]. This case report is the first reported case of erotomania underpinned by a mendelian event. This raises the question of the genetic workup in erotomania cases especially in cases with a family and/or a personal neurodevelopmental disorder.

Autism Spectrum Disorder (ASD) is characterized at the neuropsychological level by a deficit in social cognition. The concept of social cognition brings together theory of mind (being able to understand the cognitive and emotional states of others), the identification of emotions, attributional style (which can lead to interpretative biases), perception and social knowledge [45]. Two of the family members whose cases are reported here present with ASD, and two further members displayed social cognition disorders that do not fulfill the ASD diagnosis criteria.

In the literature, *AUTS2* deficient mice displayed a decrease in exploratory behavior as well as lower anxiety-like behaviors in the absence of any motor dysfunction [46]. These findings highlight how critical *AUTS2* is to the acquisition of neurocognitive function [47]. These cases not only reinforce the hypothesis of a social cognitive disorder in *AUTS2*, but more generally show that a cognitive disorder linked to a genetic mutation can lead to a range of psychiatric diagnoses. This reinforces the notion of a continuum between the psychiatric entities categorized in the DSM-5 [48], especially with regard to transdiagnostic models [49]. The precision provided by genetic analyses makes it possible to go beyond the diagnostic categories of statistical psychiatry [50].

## Conclusion

This report sheds new light on AUTS2 syndrome. The clinical and functional prognosis of individuals with a psychiatric phenotype such as ASD, ID or any other cognitive impairment depends on the early causal identification of their disorder, which is a prerequisite for adapted care.

Cognitive performance and psychiatric disorders may be more variable than previously suggested within the same family. In particular, simple social cognitive disorders might be mistaken for a delusional syndrome and/or an ASD. Identifying a gene that might relate to social cognitive disorders could be beneficial for other family members with less visible disorders.

Moreover, we have shown that a genetic event could explain a case of erotomania. The fact that a genetic mutation has been found in the context of isolated erotomania for the first time shows that we could offer genetic analysis in the context of erotomania even if the symptom is isolated.

A comprehensive clinical characterization of AUTS2 syndrome, with representations of different genetic alterations and phenotypic severity, should involve biological studies, and take into account these new data, especially with regard to the wider variability of intellectual outcomes and psychiatric disorders.

## Supplementary Information

**Additional file 1: Clinical data (supplementary materials)**.

**Additional file 2: Aunt 1**. [A] and [B]: front and side photographs. Note her heart-shaped face, soft fine hair, highly arched eyebrow, high broad forehead, bilateral ptosis, large front teeth and mild micrognathia.

**Additional file 3: Proband**. [A] and [B]: patient at 3 years of age, side and front photographs; [C] and [D]: patient at 10 years of age, side and front photographs. Note his highly arched eyebrows, hypertelorism, strabismus, proptosis and downslanted palpebral fissure, thick alae nasi, short philtrum and large central incisors, everted upper lip, narrow mouth, prominent and large ears with uptilted lobules, and retrognathia.

## Data Availability

All the data are integrated in this article. All data generated or analysed during this study are included in this published article and its supplementary information files. Thus, data sharing is not applicable to this article as no datasets were generated or analysed during the current study.

## Abbreviations

ASD: Autism spectrum disorder
*AUTS2*: AUTism susceptibility candidate 2
Array CGH: Array comparative genomic hybridization
DSM-5: Diagnostic and statistical manual, 5th ed
EEG: Electroencephalography
ICD-11: International classification of diseases, 11th ed
ID: Intellectual disability
IQ: Intellectual quotient
MRI: Magnetic resonance imaging
WAISS: Wechsler adult intelligence scale
WISC-III: Wechsler intelligence scale for children, 3th ed
