# Supplementary material for: Erotomania and phenotypic continuum in a family frameshift variant of AUTS2: a case report and review
Source: BMC Psychiatry. 2021 Jul 17;21:360. doi: 10.1186/s12888-021-03342-8 (PMC8285776; doi:10.1186/s12888-021-03342-8)
Supplement: Supplementary file 1 — Additional file 1: Clinical data (supplementary materials). [file 12888_2021_3342_MOESM1_ESM.docx]

**CLINICAL DATA (SUPPLEMENTARY MATERIALS)**

| **Authors** | Sultana et al.  (2002) (on twins of de la Barra et al. (1986) | Kalscheuer et al.  (2007) | Bakkaloglu et al.  (2008) | Huang et al.  (2010) | Nagamani et al.  (2013) | Jolley et al.  (2013) | Amarillo et al.  (2014) | Liu et al.  (2015) | Beunders et al.  (2016) | Schneider et al.  (2015) | Fan et al.  (2015) | Beunders et al.  (2015) | Sengun et al.  (2016) | Saeki et al.  (2019) |
| --- | --- | --- | --- | --- | --- | --- | --- | --- | --- | --- | --- | --- | --- | --- |
| **Dysmorphic features** | ? | + (2/3) | - | + (1/1) | + (3/4) | + (1/1) | - | - | + | + (3/3) | + (3/3) | + (2/2) | + (1/1) | + (1/1) |
| Highly arched eyebrows | na | 2/3 | - | - | na | 1/1 | - | - | na | 3/3 | 3/3 | 1/2 | 1/1 | 1/1 |
| Hypertelorism | na | 1 / 2, na | - | - | na | 1/1 | - | - | na | 3/3 | 3/3 | - | - | - |
| Proptosis | na | 2/3 | - | na | na | 1/1 | - | - | na | 1/3 | 1/3 | 1/2 | 1/1 | - |
| Short/up slanting palpebral fissures | na | 2/3 | 1/1 | na | na | 1/1 | - | - | na | 1/3 | 1/3 | 1/2 | 1/1 | - |
| Epicanthic fold | na | na | - | 1/1 | na | 0/1 | - | - | na | 1/3 | 1/3 | - | 1/1 | - |
| Strabismus | 2/2 | 1/2, na | 1/1 | na | na | 0/1 | - | - | 3/11 | 1/3 | 1/3 | 1/2 | na | - |
| Full cheek | na | 2/3 | - | na | na | 1/1 | - | - | na | 1/3 | 1/3 | na | - | - |
| Prominent nasal tip | na | - | - | 1/1 | na | 1/1 | - | - | na | - | - | 2/2 | 1.1 | - |
| Anteverted nares/thick alae | na | 2/3 | - | 1/1 | na | 1/1 | - | - | na | 3/3 | 3/3 | - | - | - |
| Narrow/deep nasal bridge | na | 2/3 | - | na | na | 1/1 | - | - | na | 3/3 | 3/3 | 2/2 | 1/1 | 1/1 |
| Short philtrum | 2/2 | 2/3 | - | 1/1 | na | 1/1 | - | - | na | 2/3 | 2/3 | 2/2 | na | - |
| Micro/retrognathia | na | 2/3 | - | 1/1 | na | 1/1 | - | - | na | 1/3 | 1/3 | 1/2 | 1/1 | 1/1 |
| Low-set ears/ Ear pit | na | 1/3 | - | 1/1 | na | 1/1 | - | - | na | 1/3 | 1/3 | 1/2 | 1/1 | 1/1 |
| Narrow mouth | 0/2 | 2/3 | - | na | na | 1/1 | - | - | 5/13 | 2/3 | 2/3 | - | 1/1 | 1/1 |
| Vermilion abnormality | na | na | - | na | na | na | na | na | na | 3/3 | 3/3 | na | na | na |
| Kyphosis/scoliosis | 2/2 | 1/3 | na | na | 1/4 | na | - | - | 3/13 | - | - | - | 1/1 | - |
| Arthrogryposis | na | 1/1, na (2) | na | na | na | na | na | - | na | - | - | 2/2 | 1/1 | - |
| Feet deformities (Pes planus) | na | na | na | na | na | na | 1/1 | na | 5/13 | 1/3 | 1/3 | - | 1/1 | na |
| Other |  | Cataract (1); Hypospadias, vesico-ureteral reflux, cryptorchidism (1) | Hearing loss | Sound sensitivity |  | Mild tremor; sound sensitivity |  |  | Myopia (2); Frequency of infection in childhood (7); eczema (4); narrow hips (5); narrow hands (7); pamprodactyly dig. V (4); Faint extension creases (6); hypersentivity (6) |  | Sound sensitivity (1/3) | Sound sensitivity (1/2) | Sound sensitivity; tight heel cords; camptodactyly; palilalia; skin pricking | Hernia umbilicalis/inguinalis |
