# Supplementary material for: Erotomania and phenotypic continuum in a family frameshift variant of AUTS2: a case report and review
Source: BMC Psychiatry. 2021 Jul 17;21:360. doi: 10.1186/s12888-021-03342-8 (PMC8285776; doi:10.1186/s12888-021-03342-8)
Supplement: Supplementary file 2 — Additional file 2: Aunt 1. [A] and [B]: front and side photographs. Note her heart-shaped face, soft fine hair, highly arched eyebrow, high broad forehead, bilateral ptosis, large front teeth and mild micrognathia. [file 12888_2021_3342_MOESM2_ESM.docx]

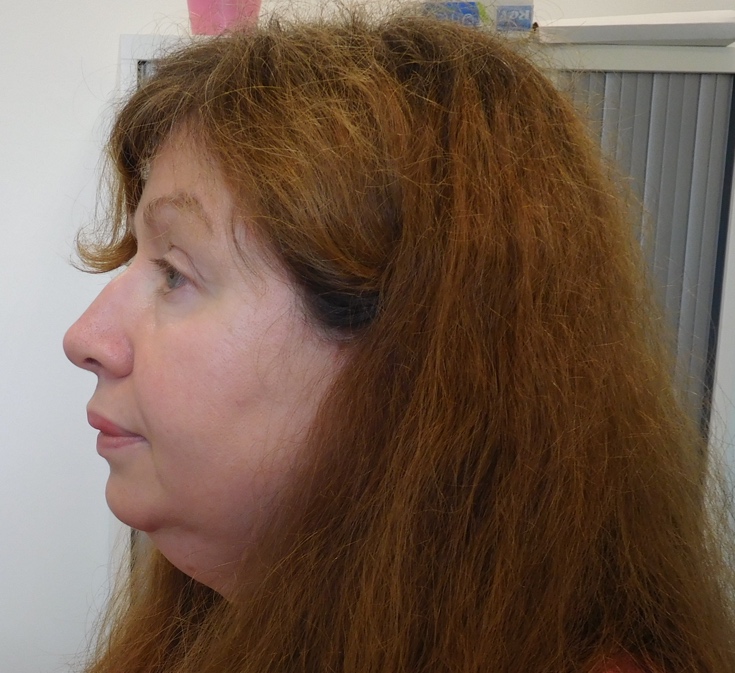

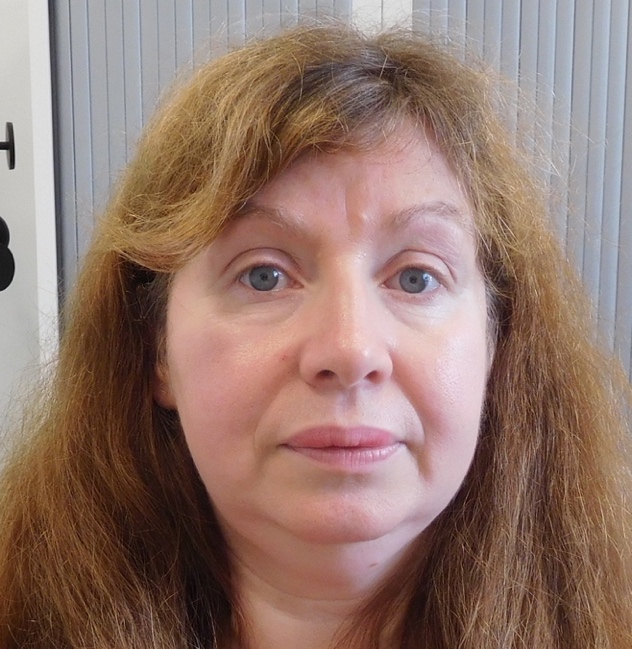


1. B.

**Aunt 1**. [A] and [B]: front and side photographs. Note her heart-shaped face, soft fine hair, highly arched eyebrow, high broad forehead, bilateral ptosis, large front teeth and mild micrognathia.
