# Supplementary material for: Erotomania and phenotypic continuum in a family frameshift variant of AUTS2: a case report and review
Source: BMC Psychiatry. 2021 Jul 17;21:360. doi: 10.1186/s12888-021-03342-8 (PMC8285776; doi:10.1186/s12888-021-03342-8)
Supplement: Supplementary file 3 — Additional file 3: Proband. [A] and [B]: patient at 3 years of age, side and front photographs; [C] and [D]: patient at 10 years of age, side and front photographs. Note his highly arched eyebrows, hypertelorism, strabismus, proptosis and downslanted palpebral fissure, thick alae nasi, short philtrum and large central incisors, everted upper lip, narrow mouth, prominent and large ears with uptilted lobules, and retrognathia. [file 12888_2021_3342_MOESM3_ESM.docx]

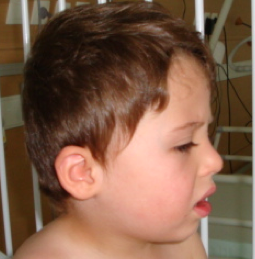

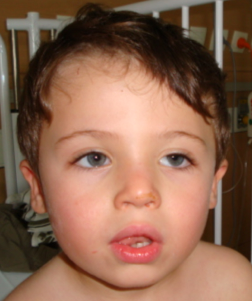

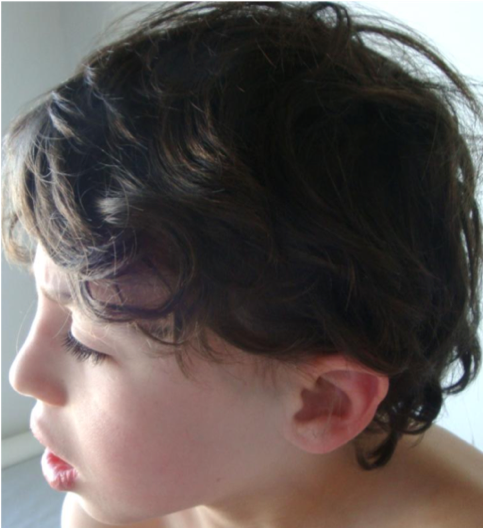

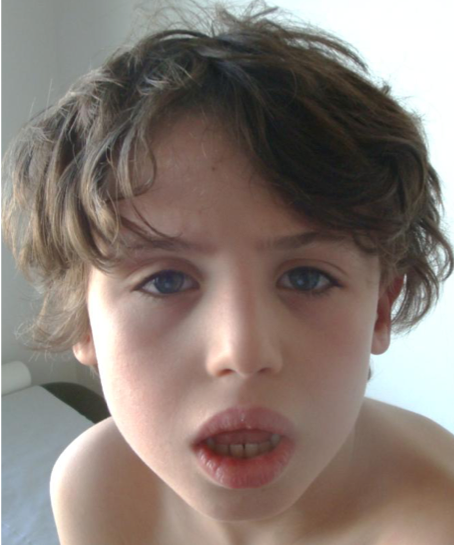


1. B. C. D.

**Proband**. [A] and [B]: patient at 3 years of age, side and front photographs; [C] and [D]: patient at 10 years of age, side and front photographs. Note his highly arched eyebrows, hypertelorism, strabismus, proptosis and downslanted palpebral fissure, thick alae nasi, short philtrum and large central incisors, everted upper lip, narrow mouth, prominent and large ears with uptilted lobules, and retrognathia.
